# A Se···O bonding catalysis approach to the synthesis of calix[4]pyrroles

**DOI:** 10.3762/bjoc.18.36

**Published:** 2022-03-18

**Authors:** Qingzhe Tong, Zhiguo Zhao, Yao Wang

**Affiliations:** 1 School of Chemistry and Chemical Engineering, Key Laboratory of the Colloid and Interface Chemistry, Shandong University, Jinan 250100, China

**Keywords:** calix[4]pyrrole, chalcogen bonding, ketones, Se···O bonding interactions, supramolecular catalysis

## Abstract

Described herein is a chalcogen bonding catalysis approach to the synthesis of calix[4]pyrrole derivatives. The Se···O bonding interactions between selenide catalysts and ketones gave rise to the catalytic activity in the condensation reactions between pyrrole and ketones, leading to the generation of calix[4]pyrrole derivatives in moderate to high yields. This chalcogen bonding catalysis approach was efficient since only 5 mol % catalyst loading was used to promote the consecutive condensation processes while the reactions could be carried out at room temperature, thus highlighting the potential of this type of nonclassical interactions in catalyzing relative complex transformations.

## Introduction

Noncovalent catalysis has been established as one of the fundamental concepts in organic synthesis that enables achieving numerous chemical transformations [1]. Among these noncovalent forces, hydrogen bonding interactions play a central role in noncovalent catalysis [2] while halogen bonding interactions have lately been exploited as a new tool to catalyze a diverse array of reactions [3–5]. In addition, nonclassical interactions such as anion–π [6–11] as well as chalcogen [12–17] and pnictogen [18–23] bonds were established as emerging driving forces for the development of organic reactions. Very recently, catalysis with carbon bonding interactions was realized and this type of catalysis mode was capable of facilitating a range of typical reactions [24], thus providing a new platform for organic synthesis.

The phenomenon of chalcogen bonding was initially observed in the crystal structures of small organic molecules as well as proteins [25]. The application of this type of bonding interactions has achieved significant advances in the research fields of crystal engineering [26], medicinal chemistry [27], anion recognition [28–32] and transport [33–35]. In addition, intramolecular chalcogen bonding interactions have been suggested to stabilize reactive intermediates in a range of isothiourea-catalyzed transformations, which play a key factor to modulate the selectivity of these reactions [36–40]. In addition, few examples demonstrated that disubstituted chalcogens could be used as effective catalysts through intermolecular chalcogen bonding interactions [41–49]. Despite these significant advances, catalysis with chalcogen bonding interactions is still in its infancy and the development of new types of reactions is highly desirable.

**Scheme 1:**
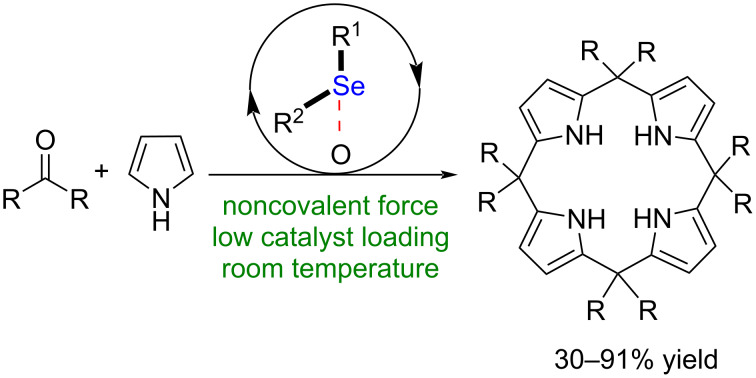
Proposal of a Se···O bonding catalysis approach.

Calix[4]pyrrole derivatives have been widely used as transition metal ligands and functional materials [50–53]. Thus far, several synthetic methods to access these compounds have been reported [54–55]. The classical approaches to synthesis of calix[4]pyrrole derivatives mainly involved a stepwise synthesis and Lewis acid as well as Brønsted acid catalysis [54–55]. Notably, a noncovalent catalysis approach to accessing calix[4]pyrrole derivatives remains underdeveloped. To provide a new strategy to synthesize calix[4]pyrrole derivatives, herein, we describe a Se···O bonding catalysis approach to accessing this type of compounds (Scheme 1).

## Results and Discussion

### Evaluation of catalysts

**Scheme 2:**
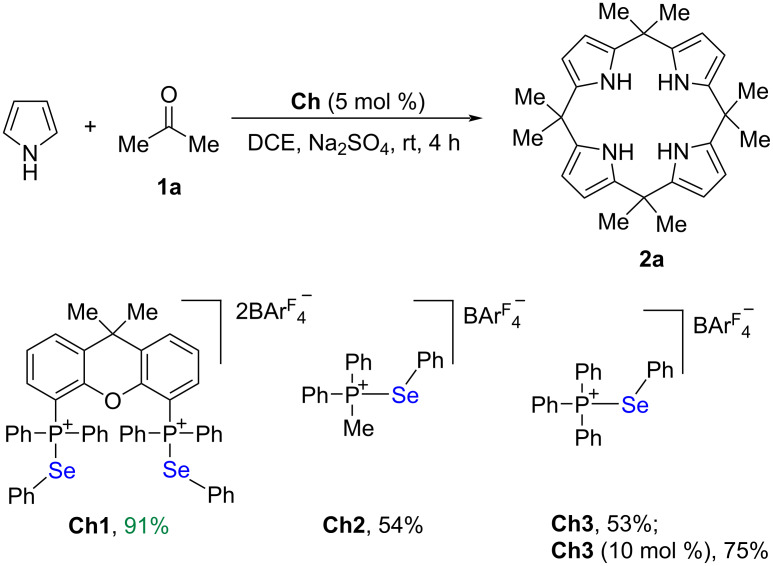
Se···O bonding catalysis approach to the synthesis of calix[4]pyrrole **2a**.

We developed a class of phosphonium selenide catalysts which showed catalytic activity in assembly reactions [41], Michael addition reactions [41], Rauhut–Currier reactions [42], cyanosilylation reactions [43], and cycloaddition of vinylindoles through chalcogen–π bonding catalysis [44]. Our previous works demonstrated that Se···O bonding interactions between phosphonium selenides and carbonyls can significantly activate carbonyl groups [41–43], thus providing a new opportunity to develop carbonyl chemistry. To expand the catalysis capability of chalcogen bonding interactions, we envisioned that consecutive condensations between ketones and pyrrole might take place to give calix[4]pyrrole derivatives under catalysis of a selenide catalyst. In the absence of a catalyst, no reaction took place. Indeed, even in presence of 5 mol % representative catalyst **Ch1** [44], the condensation reaction between acetone and pyrrole worked efficiently at room temperature. We note that this reaction did not stop at a bis(pyrrole)methane stage, but consecutive condensations between four molecules of acetone and four molecules of pyrrole took place to give calix[4]pyrrole **2a** in 91% yield after 4 h (Scheme 2). Further investigations revealed that the monodentate catalysts were less active and only a moderate yield was obtained regardless of whether **Ch2** or **Ch3** was used. In the presence of 10 mol % catalyst **Ch3**, 75% yield of **2a** was obtained.

### Reaction scope

**Scheme 3:**
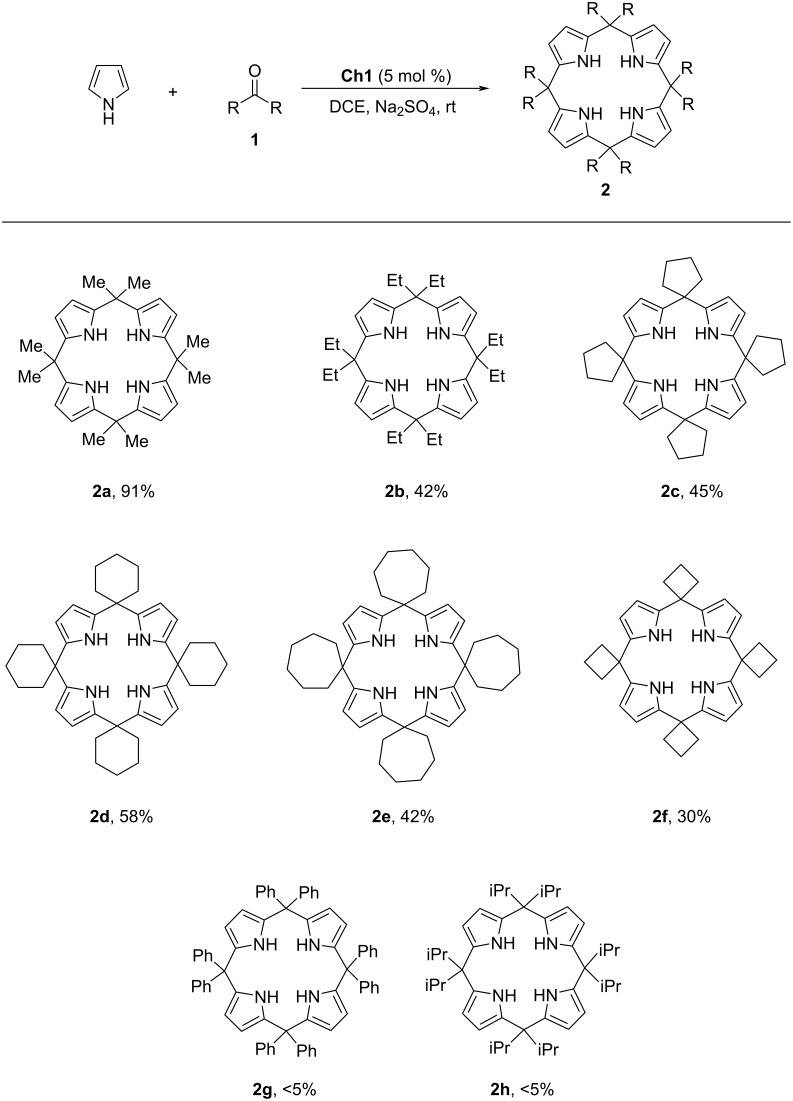
Reaction scope.

Inspired by the good result obtained with catalyst **Ch1**, the scope of ketones was investigated (Scheme 3). Both linear and cyclic aliphatic ketones could be used to synthesize calix[4]pyrrole derivatives under catalysis of 5 mol % **Ch1** at room temperature. It was found that this chalcogen bonding catalysis approach was susceptible to the variation of the steric environment of ketones. Upon changing acetone to pentan-3-one, the chemical yield decreased significantly and product **2b** was obtained in 42% yield. Using cyclopentanone as a reactant, product **2c** was obtained in 45% yield. Moreover, cyclohexanone and cycloheptanone could also be used as effective reactants, and products **2d** and **2e** were obtained in 58% and 42% yield, respectively. Further investigation revealed that cyclobutanone was reactive in this transformation to give product **2f**, albeit with 30% yield. However, benzophenone and 2,4-dimethylpentan-3-one with high steric hindrance failed to give desirable products **2g** and **2h**. Further investigation on using an asymmetric ketone such as pentan-2-one as a reactant showed that the reaction gave an inseparable mixture of diastereomers. Meanwhile, upon using benzaldehyde as a reactant, the reaction system was complex and there was no major product.

### Proposed activation mode

**Scheme 4:**
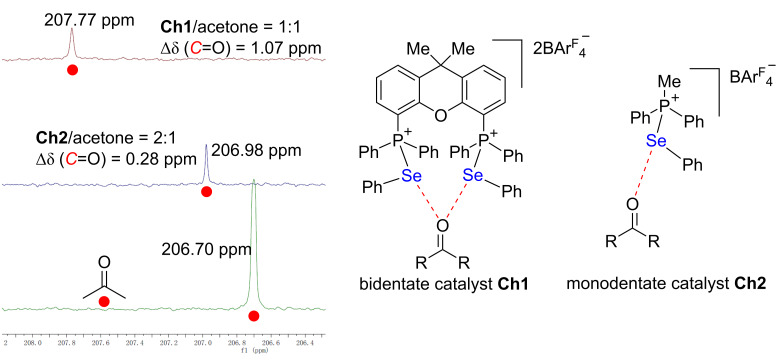
Proposed activation mode.

The chalcogen bonding interactions between catalysts **Ch1**, **Ch2** and acetone were examined by ^13^C NMR experiments in CD_2_Cl_2_. The interaction between bidentate catalyst **Ch1** or monodentate catalyst **Ch2** and acetone could result in a variation of the ^13^C signal of the carbonyl group. Analysis of a 1:1 mixture of **Ch1** and acetone in CD_2_Cl_2_ indicated a 1.07 ppm downfield shift of the ^13^C signal of the carbonyl group, while a 0.28 ppm downfield shift of the ^13^C signal of the carbonyl group was observed upon analysis of a 2:1 mixture of **Ch2** and acetone (Scheme 4). Therefore, in line with the catalytic results as depicted in Scheme 2, both monodentate and bidentate catalysts could activate ketones. Accordingly, either a single activation or a double activation mode could be an effective driving force to promote this transformation, albeit with distinct catalytic activity.

## Conclusion

In summary, we developed a Se···O bonding catalysis approach to the synthesis of calix[4]pyrroles. In the presence of 5 mol % selenide catalyst, calix[4]pyrrole products were obtained in moderate to good yields at room temperature. The experimental results showed that both bidentate and monodentate catalysts were catalytically active in the condensation reactions between pyrrole and ketones. In addition, both cyclic and linear aliphatic ketones were effective reactants in this transformation. This work provides a new strategy to access calix[4]pyrrole derivatives and makes an important complementation to the research topic of chalcogen bonding catalysis.

## Supporting Information

File 1 Full experimental procedures and compound characterization.
