# Supplementary material for: A Se···O bonding catalysis approach to the synthesis of calix[4]pyrroles
Source: Beilstein J Org Chem. 2022 Mar 18;18:325–30. doi: 10.3762/bjoc.18.36 (PMC8941317; doi:10.3762/bjoc.18.36)

BEILSTEIN JOURNAL OF ORGANIC CHEMISTRY

Supporting Information

for

A Se···O bonding catalysis approach to the synthesis of calix[4]pyrroles

Qingzhe Tong, Zhiguo Zhao and Yao Wang

Beilstein J. Org. Chem. **2022,** *18,* 325–330. doi:10.3762/bjoc.18.36

Full experimental procedures and compound characterization

License and Terms: This is a supporting information file under the terms of the Creative Commons Attribution License (https://creativecommons.org/licenses/by/4.0). Please note that the reuse, redistribution and reproduction in particular requires that the author(s) and source are credited and that individual graphics may be subject to special legal provisions.
The license is subject to the *Beilstein Journal of Organic Chemistry* terms and conditions: (https://www.beilstein-journals.org/bjoc/terms)

Table of Contents:

1. General information ..…………………………….……………………………….S2

2. General experimental procedure.……….……..….….…………........…...….S2–S3

3. Analytical data………………..…………...…...…………………..…..…S3–S5

4. Copies of NMR spectra…………………………..…………..…….............S6–S11

1. General information

All the chemicals were either purchased from commercial suppliers or purified by standard procedures as specified in *Purification of Laboratory Chemicals*, 7th Ed (Armarego, W. L. F.; Chai, C. L. L. Butterworth Heinemann: 2013). All reactions were carried out under argon atmosphere. Analytical thin-layer chromatography (TLC) was performed on silica gel plates and analyzed by UV light or by potassium permanganate or vanillin sulfuric acid stains followed by heating. Flash chromatography was carried out utilizing silica gel (200–300 mesh). ^{1}H NMR, ^{13}C NMR spectra were recorded in $CDCl_3$ at room temperature on a Bruker AM-400 spectrometer (400 MHz ^{1}H, 100 MHz ^{13}C). The chemical shifts are reported in ppm relative to either the residual solvent peak (^{13}C) (δ = 77.00 ppm for $CDCl_3$; δ = 53.84 ppm for CD_2Cl_2), (^{1}H) (δ = 7.26 ppm for $CDCl_3$ or TMS (^{1}H) (δ = 0 ppm) as an internal standard. Data for ^{1}H NMR are reported as follows: chemical shift (δ ppm), multiplicity (s = singlet, d = doublet, t = triplet, m = multiplet, dd = doublet doublet), coupling constant (Hz), integration. Data for ^{13}C NMR are reported as chemical shift. HRMS were performed on a Bruker Apex II mass instrument (ESI).

2. General experimental procedure

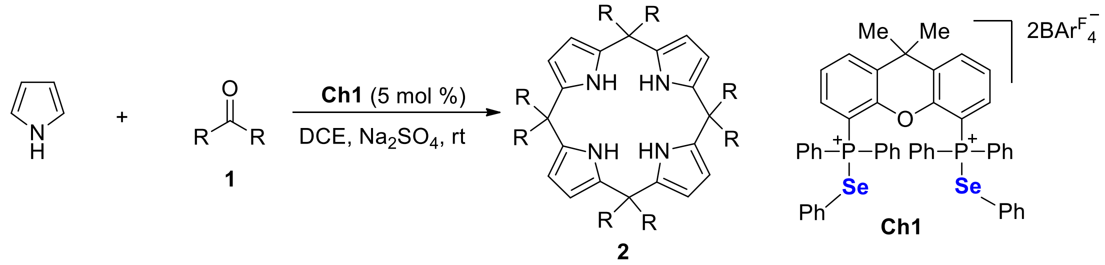

Catalysts **Ch1–3** were prepared according to the reported procedure (Kong, X.; Zhou, P.-P.; Wang, Y. *Angew. Chem., Int. Ed.* **2021**, *60*, 9395-9400.

To a dried 10 mL Schlenk tube equipped with magnetic bar was added **Ch1** (26.3 mg, 0.01 mmol) and Na_2SO_4 (50.0 mg). Then DCE (0.5 mL), pyrrole (14.0 μL, 0.2 mmol) and ketone (0.3 mmol) were added under an argon atmosphere. This reaction mixture was stirred at room temperature until the reaction was complete as judged by TLC analysis. Then the solvent was removed and the residue was purified by column chromatography (PE/EA = 120:1 to 50:1) to afford the calix[4]pyrrole product.

3. Analytical data

calix[4]pyrrole **2a**:

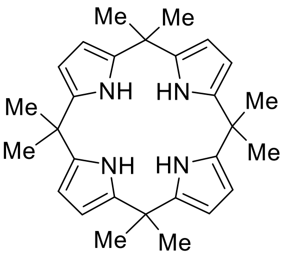

2a

Compound **2a** was synthesized according to the general procedure as a white solid in 91% yield; **^{1}H NMR** (400 MHz, $CDCl_3$): δ 1.51 (s, 24H), 5.90 (d, *J* = 2.8 Hz, 8H), 7.01-7.06 (brs, 4H); **^{13}C NMR** (100 MHz, $CDCl_3$): δ 29.08, 35.17, 102.81, 138.40; **HRMS** (ESI+): exact mass calculated for $[M+H]^+$ ($C_{28}H_{37}N_4$) requires m/z 429.3018, found: m/z 429.3015. Compound **2a** is known in the literature and the above spectral data are in agreement with the literature report (Nishiyabu, R.; Palacios, M. A.; Dehaen, W.; Anzenbacher, P. *J. Am. Chem. Soc.* **2006**, *128*, 11496-11504).

calix[4]pyrrole **2b**:

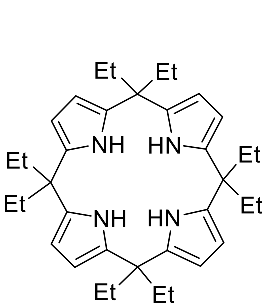

2b

Compound **2b** was synthesized according to the general procedure as a white solid in 42% yield; **^{1}H NMR** (400 MHz, $CDCl_3$): δ 0.59 (t, *J* = 7.4 Hz, 24H), 1.62-1.93 (brs, 16H), 5.90 (d, *J* = 2.8 Hz, 8H), 6.95-7.01 (brs, 4H); **^{13}C NMR** (100 MHz, $CDCl_3$): δ 8.22, 29.23, 43.12, 105.14, 135.98;

HRMS (ESI+): exact mass calculated for $[M+H]^+$ ($C_{36}H_{53}N_4$) requires m/z 541.4265, found: m/z 541.4262. Compound **2b** is known in the literature and the above spectral data are in agreement with the literature report (Mohebbi, S.; Rayati, S. *Transition Met. Chem. (Dordrecht, Neth.)* **2007**, *32*, 1035-1038).

calix[4]pyrrole **2c**:

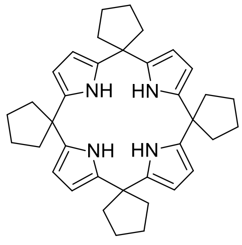

2c

Compound **2c** was synthesized according to the general procedure as a white solid in 45% yield; **^{1}H NMR** (400 MHz, $CDCl_3$): δ 1.62-1.75 (m, 16H), 2.01-2.03 (m, 16H), 5.85 (d, *J* = 2.8 Hz, 8H), 6.90-7.13 (brs, 4H); **^{13}C NMR** (100 MHz, $CDCl_3$): δ 23.84, 39.00, 46.86, 103.02, 137.06; **HRMS** (ESI+): exact mass calculated for $[M+H]^+$ ($C_{36}H_{45}N_4$) requires m/z 533.3644, found: m/z 533.3647. Compound **2c** is known in the literature and the above spectral data are in agreement with the literature report (Rawat, A. K.; Chauhan, S. M. S. *Tetrahedron Lett.* **2014**, *55*, 6969-6971).

calix[4]pyrrole **2d**:

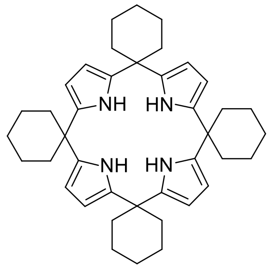

2d

Compound **2d** was synthesized according to the general procedure as a white solid in 58% yield; **^{1}H NMR** (400 MHz, $CDCl_3$): δ 1.36-1.54 (m, 24H), 1.88-1.96 (m, 16H), 5.90 (d, *J* = 2.7 Hz, 8H), 7.01-7.07 (brs, 4H); **^{13}C NMR** (100 MHz, $CDCl_3$): δ 22.75, 26.02, 37.23, 39.63, 103.49, 136.42; **HRMS** (ESI+): exact mass calculated for $[M+H]^+$ ($C_{36}H_{53}N_4$) requires m/z 589.4265, found: m/z 589.4262. Compound **2d** is known in the literature and the above spectral data are in agreement with the literature report (Jain, V. K.; Mandalia, H. C.; Suresh, E. *J. Inclusion Phenom. Macrocyclic Chem.* **2008**, *62*, 167-178).

calix[4]pyrrole **2e**:

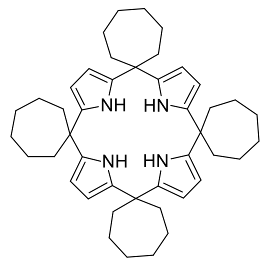

2e

Compound **2e** was synthesized according to the general procedure as a white solid in 42% yield; **^{1}H NMR** (400 MHz, $CDCl_3$): 1.52-1.56 (m, 32H), 2.00-2.03 (m, 16H), 5.87 (d, *J* = 2.8 Hz, 8H), 6.86-6.93 (brs, 4H); **^{13}C NMR** (100 MHz, $CDCl_3$): δ 23.15, 29.76, 39.39, 42.73, 102.95, 138.30; **HRMS** (ESI+): exact mass calculated for $[M+H]^+$ ($C_{36}H_{61}N_4$) requires m/z 645.4891, found: m/z 645.4884.

calix[4]pyrrole **2f**:

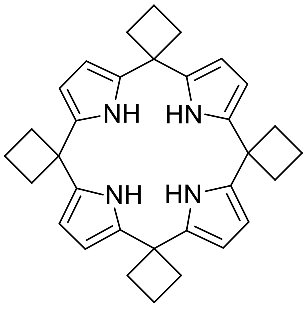

2f

Compound **2f** was synthesized according to the general procedure as a white solid in 30% yield; **^{1}H NMR** (400 MHz, $CDCl_3$): δ 1.97 (q, *J* = 8.1 Hz, 8H), 2.41 (t, *J* = 7.7 Hz, 16H), 6.06 (d, *J* = 2.8 Hz, 8H), 6.79-6.87 (brs, 4H); **^{13}C NMR** (100 MHz, $CDCl_3$): δ 16.47, 34.51, 41.27, 104.00, 137.23; **HRMS** (ESI+): exact mass calculated for $[M+H]^+$ ($C_{36}H_{37}N_4$) requires m/z 477.3013, found: m/z 477.3015.

4. Copies of NMR spectra

^{1}H NMR spectrum of compound **2a** ($CDCl_3$, 400 MHz):

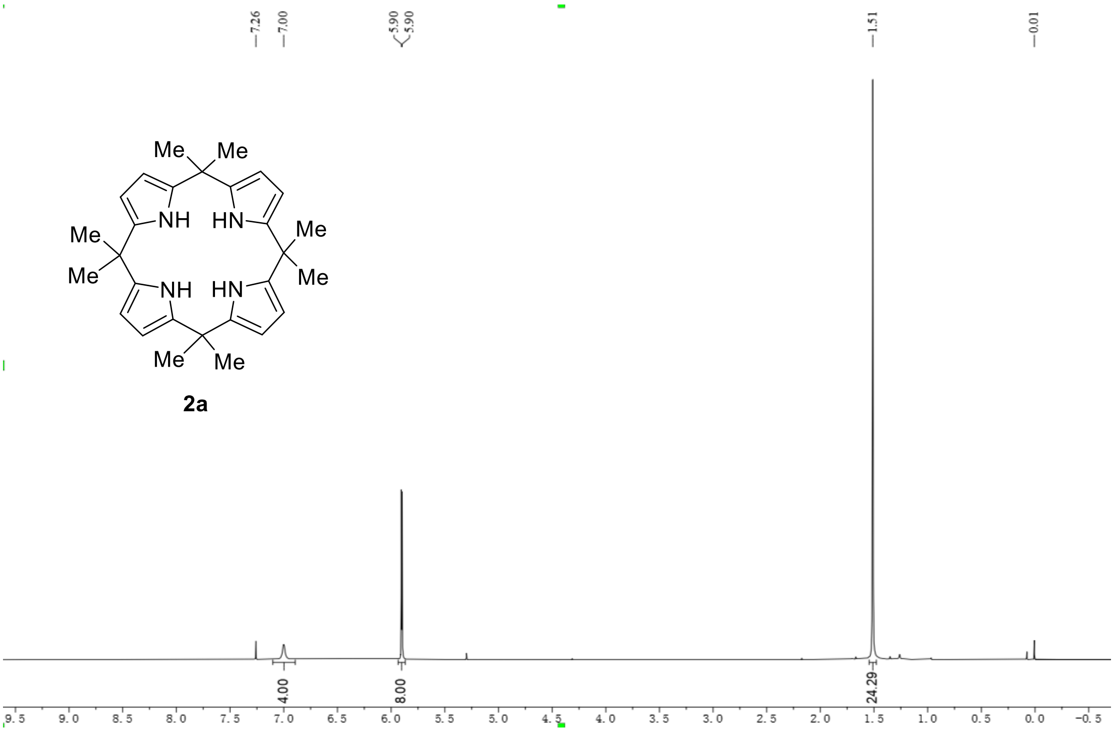

^{13}C NMR spectrum of compound **2a** ($CDCl_3$, 100 MHz):

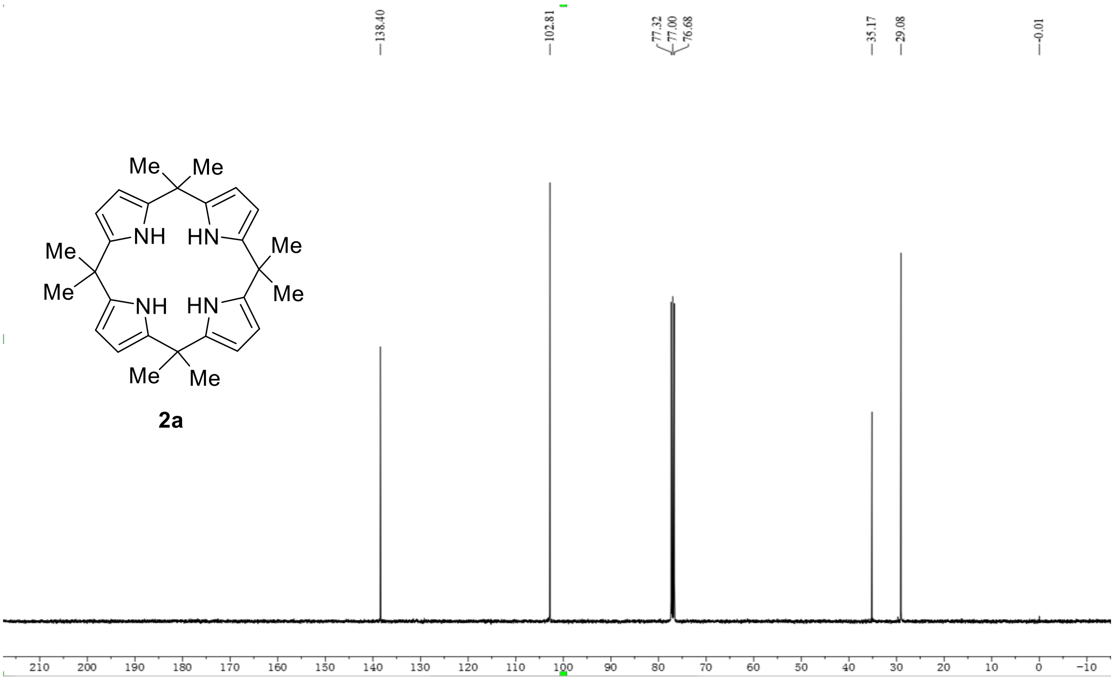

^{1}H NMR spectrum of compound **2b** ($CDCl_3$, 400 MHz):

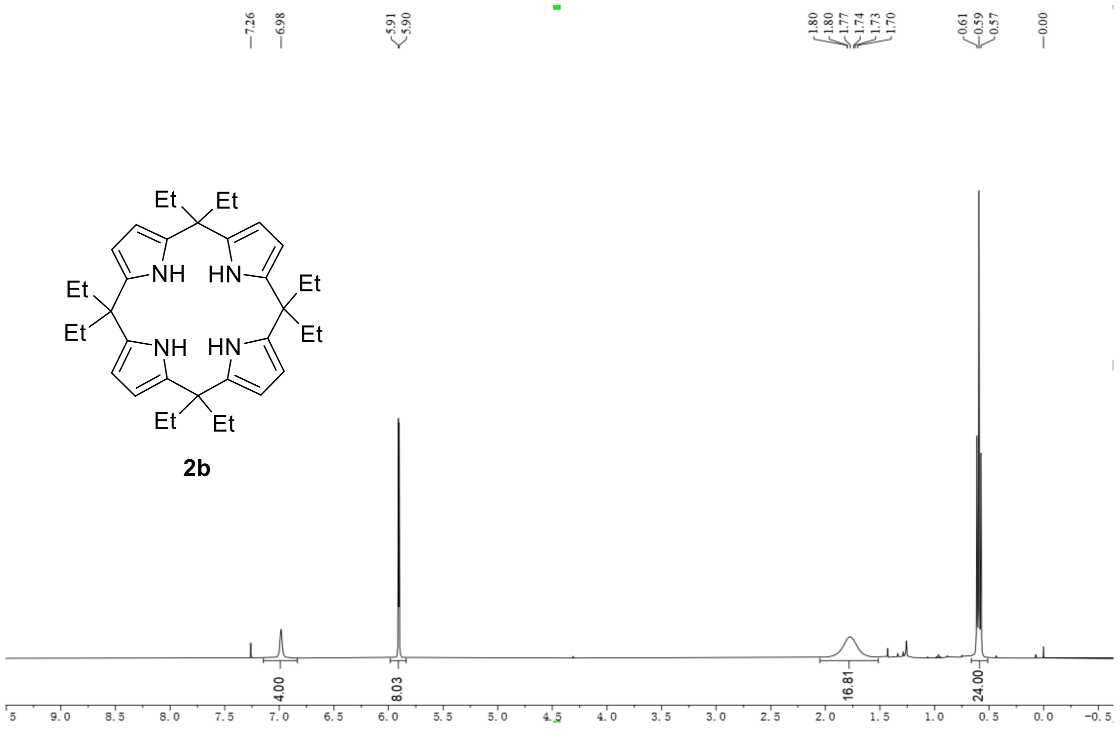

^{13}C NMR spectrum of compound **2b** ($CDCl_3$, 100 MHz):

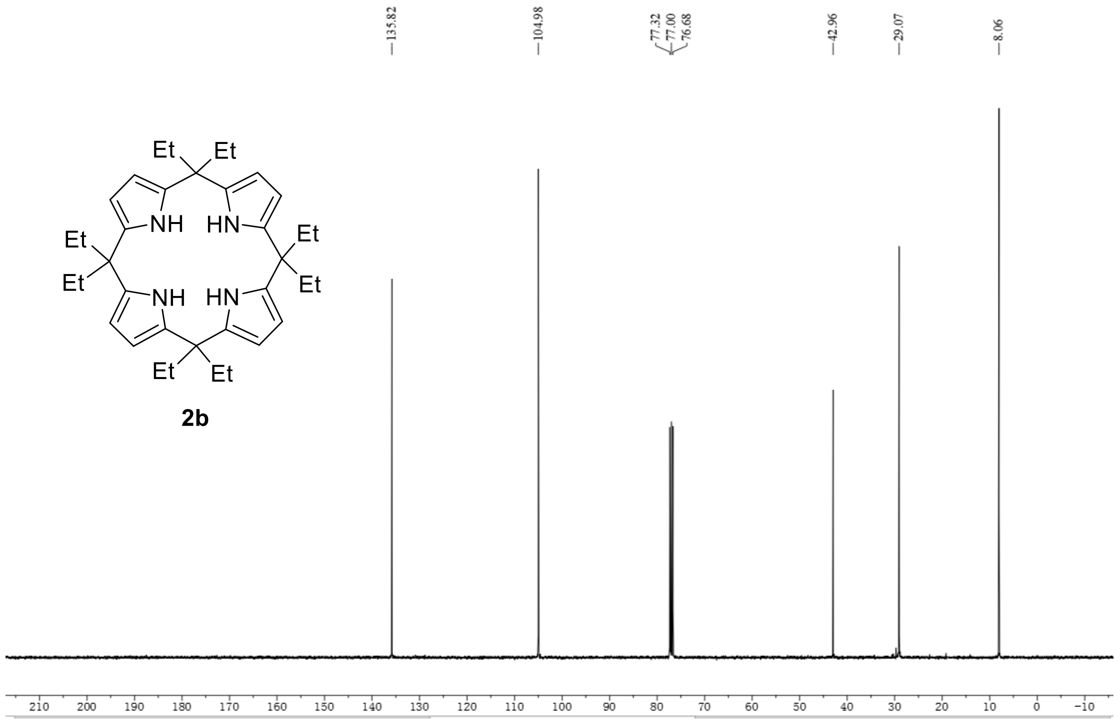

^{1}H NMR spectrum of compound **2c** ($CDCl_3$, 400 MHz):

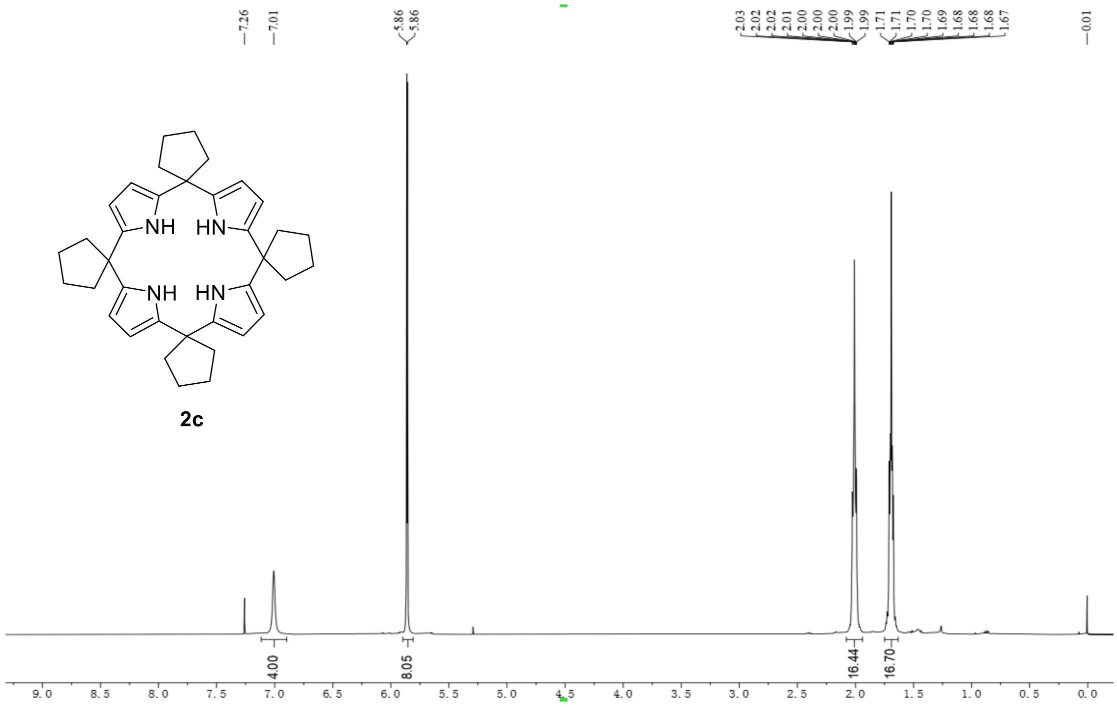

^{13}C NMR spectrum of compound **2c** ($CDCl_3$, 100 MHz):

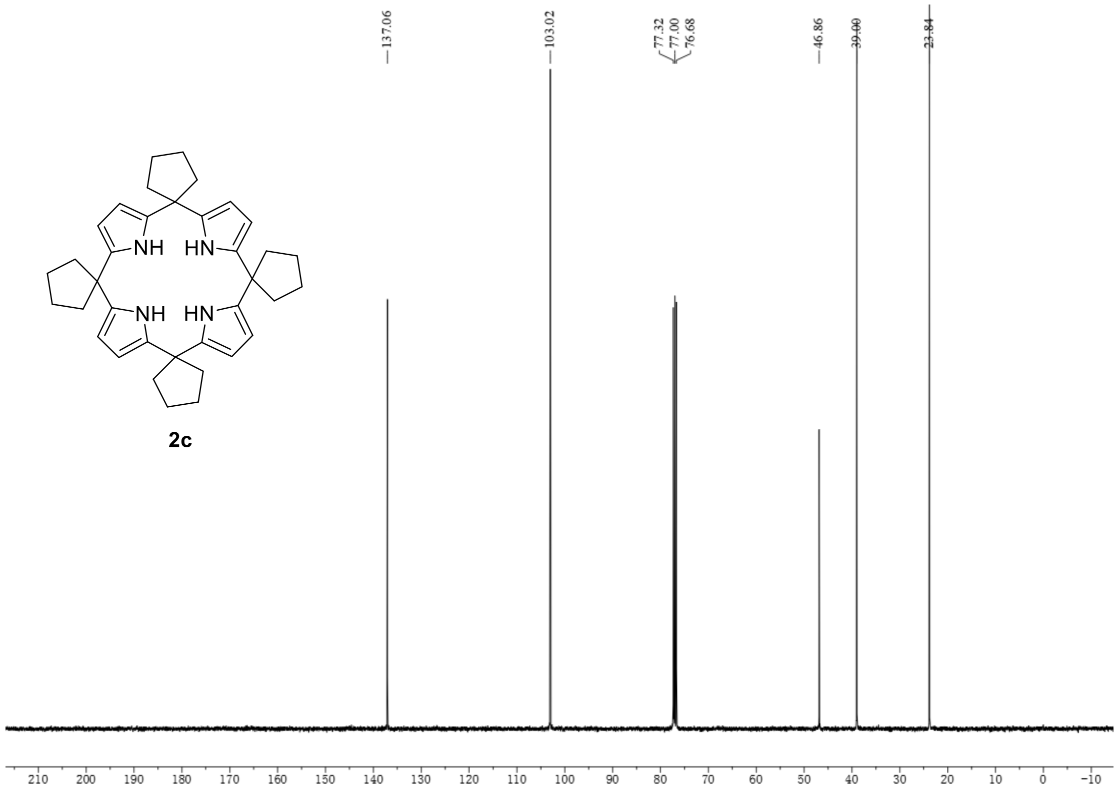

[1]**H NMR** spectrum of compound **2d** ($CDCl_3$, 400 MHz):

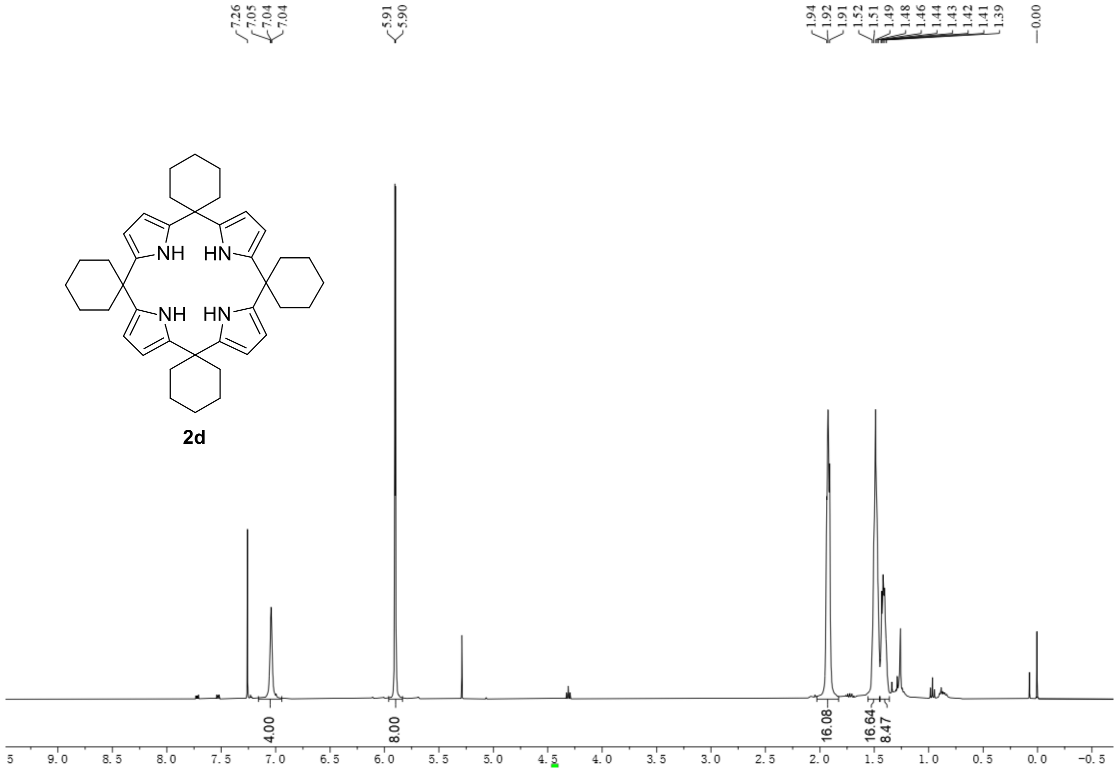

[13]**C NMR** spectrum of compound **2d** ($CDCl_3$, 100 MHz):

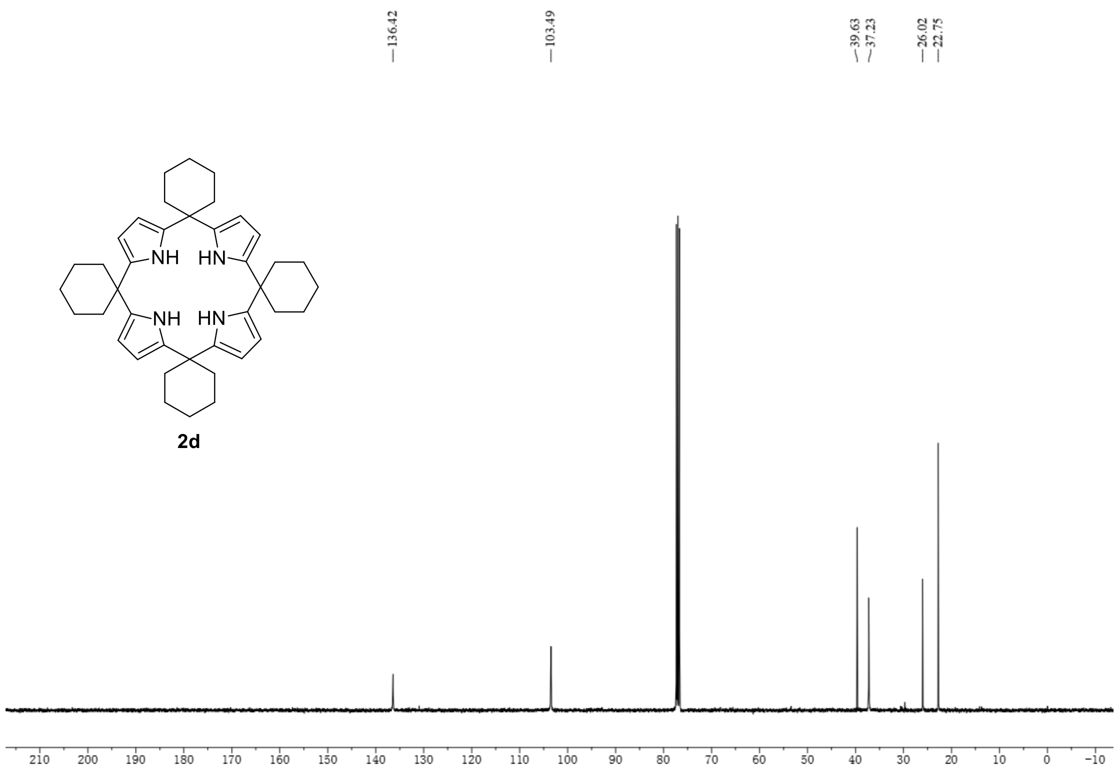

^{1}H NMR spectrum of compound **2e** ($CDCl_3$, 400 MHz):

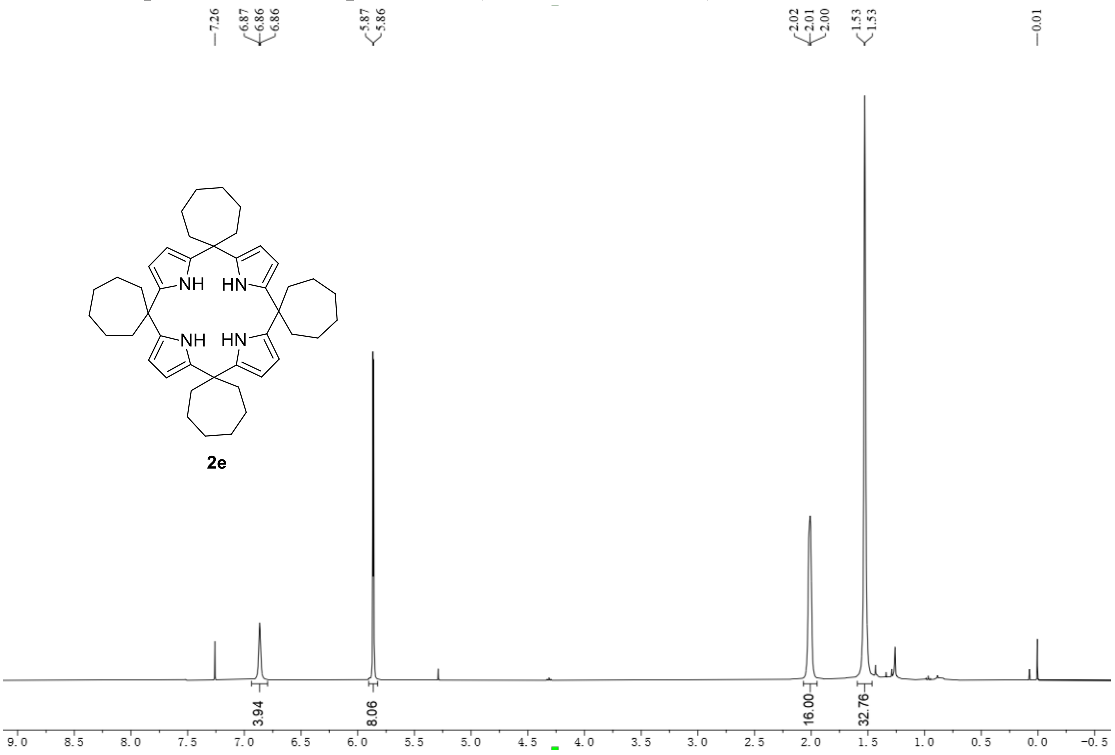

^{13}C NMR spectrum of compound **2e** ($CDCl_3$, 100 MHz):

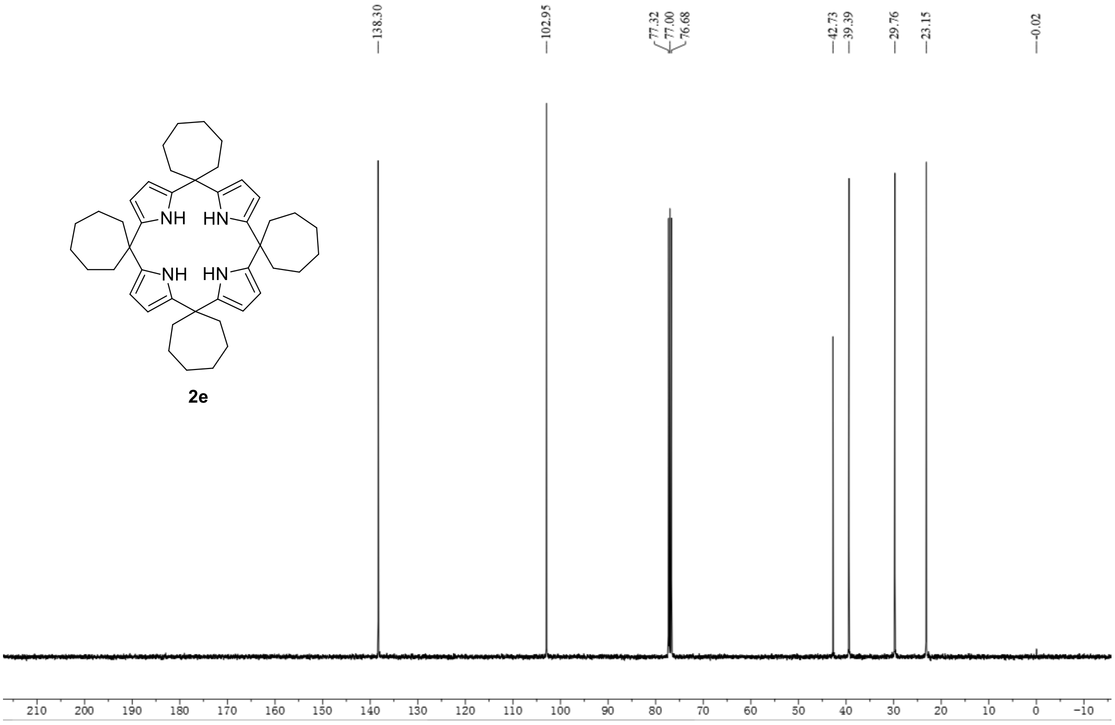

^{1}**H NMR** spectrum of compound **2f** ($CDCl_3$, 400 MHz):

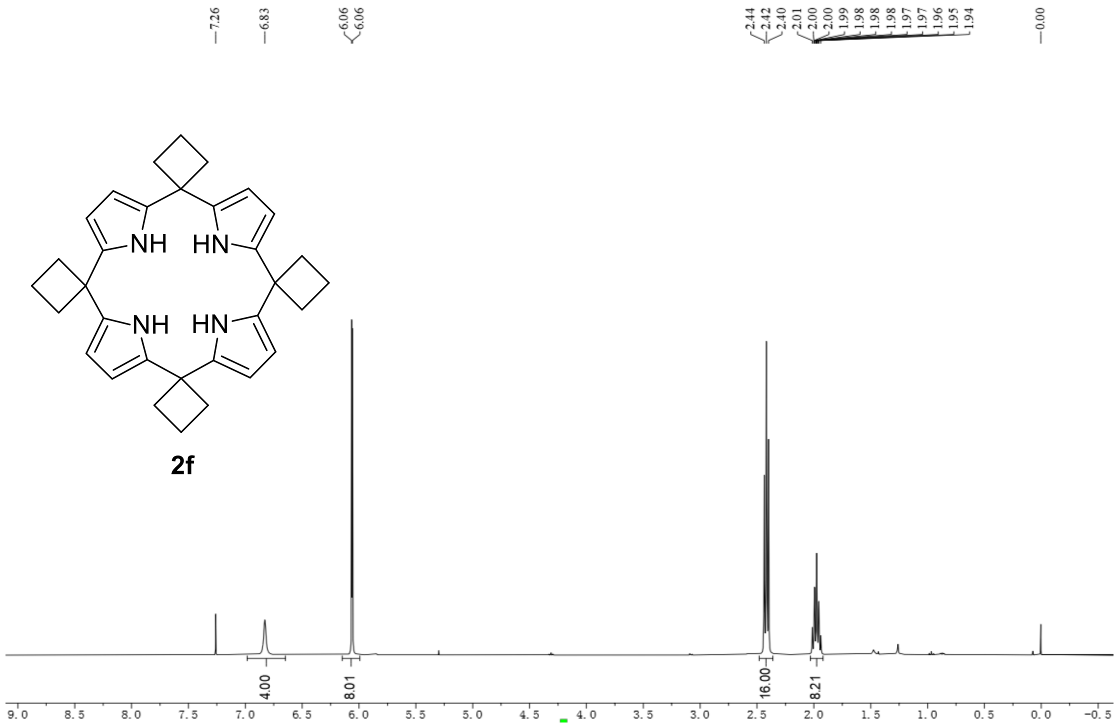

^{13}**C NMR** spectrum of compound **2f** ($CDCl_3$, 100 MHz):

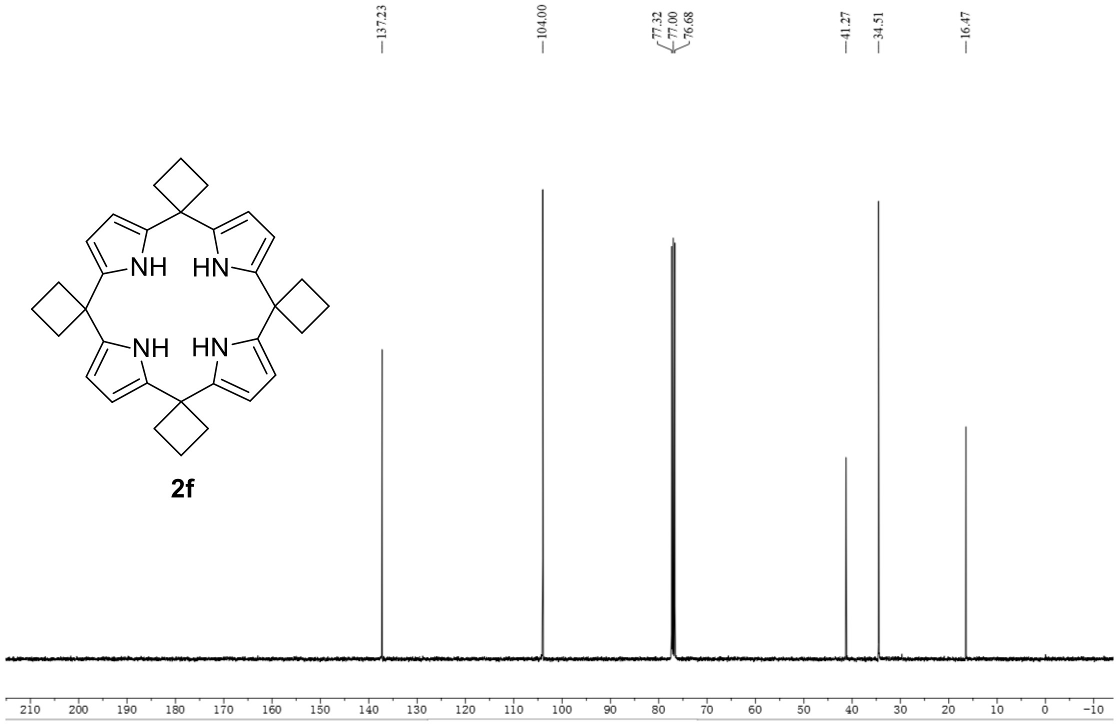

Supplement: File 1 — Full experimental procedures and compound characterization. [file Beilstein_J_Org_Chem-18-325-s001.pdf]
